# Retained Gallstone Presenting as Large Intra-abdominal Mass Four Years after Laparoscopic Cholecystectomy

**DOI:** 10.7759/cureus.2030

**Published:** 2018-01-06

**Authors:** Gabriel O Ologun, Rachel Lovely, Mohammad Sultany, Mustafa Aman

**Affiliations:** 1 General Surgery, Robert Packer Hospital/Guthrie Clinic; 2 Medical Student, Geisinger Commonwealth School of Medicine/robert Packer Hospital; 3 Minimally Invasive/bariatric Surgery, Robert Packer Hospital/Guthrie Clinic

**Keywords:** abdominal mass, retained gallstones, spilt gallstone, peritoneal gallstone, laparoscopic cholecystectomy

## Abstract

Gallstone spillage is a common intraoperative event during laparoscopic cholecystectomy (LC). Patients present months to years with nonspecific symptoms after the original procedure. The complications of retained peritoneal gallstones are infrequent. Having a high index of suspicion is the key to early diagnosis. Every effort should be made to remove spilled gallstones at the index operation to prevent future complications, however, conversion from laparoscopy to laparotomy for retrieval of spilt gallstone is not recommended. Here we present a case of retained gallstone presenting as a large intra-abdominal mass four years after laparoscopic cholecystectomy in a middle age bariatric patient.

## Introduction

Laparoscopic cholecystectomy (LC) is the treatment of choice for acute cholecystitis as well as for symptomatic cholelithiasis. Gallstone spillage is a common intraoperative event during LC. Woodfield, et al. report an incidence of gallbladder perforation of 18.3%, with complication of peritoneal gallstones occurring in approximately 1.7 per 1,000 LCs, in approximately 2.3% of cases in which stones were spilt, and in 7% of cases in which stones were knowingly left unretrieved in the peritoneal cavity [1]. Patients present months to years with nonspecific symptoms after the original procedure [1,2]. These complications include intra-abdominal abscess, abdominal wall abscess (including port site abscess), peritonitis, ileus, small bowel obstruction, small bowel fistula, bowel perforation, and colonic fistula [1-5]. Here we present a case of retained gallstone presenting as a large intra-abdominal mass four years after laparoscopic cholecystectomy in a middle age bariatric patient. Informed consent was obtained for the case report, images, and for publication.

## Case presentation

A 52-year-old female presented in our bariatric clinic for routine follow-up for history of laparoscopic sleeve gastrectomy about five years ago for morbid obesity who reported having occasional postprandial epigastric pain that had been ongoing for about three months. There was no associated nausea or vomiting. She was not on antacid or proton pump inhibitor. History was significant for laparoscopic cholecystectomy four years ago for biliary colic with iatrogenic perforation of the gallbladder due to dense adhesion, all visible gallstones were aspirated at that time and her recovery was uneventful. She had also had diagnostic laparoscopy, laparoscopic-assisted hysterectomy and bilateral salpingo-oophorectomy, cesarean-section. She was on multivitamins at home. Physical examination was unremarkable – she weighed 106 kg, height 1.72 m with a body mass index (BMI) of 35.8 kg/m^2^. Blood tests revealed vitamin B1 (thiamine) and Vitamin D (cobalamin) deficiency, the rest of the laboratory results were within normal limits.

**Figure 1:**
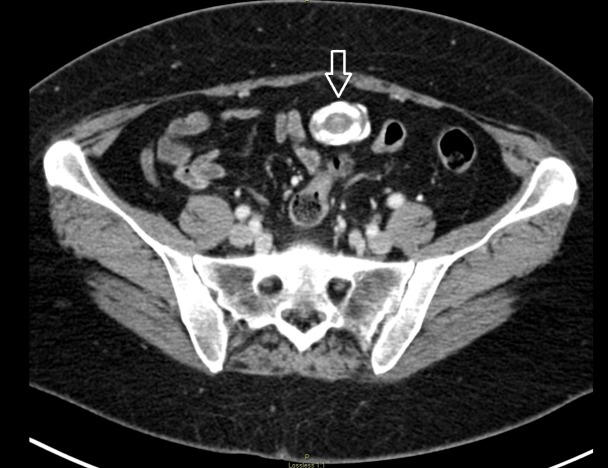
Computed tomography scan of abdomen and pelvis axial view showing calcified intra-abdominal mass (arrow).

On this occasion, upper endoscopy was done which showed small amount of bile gastritis, otherwise, it was unremarkable. Subsequent computed tomography (CT) scan revealed a densely calcified lesion within the omentum without surrounding inflammation (Figure 1). The patient underwent diagnostic laparoscopy, and laparoscopic resection of intra-abdominal mass. Intraoperatively a large, firm mass was found encased in the omentum.

**Figure 2:**
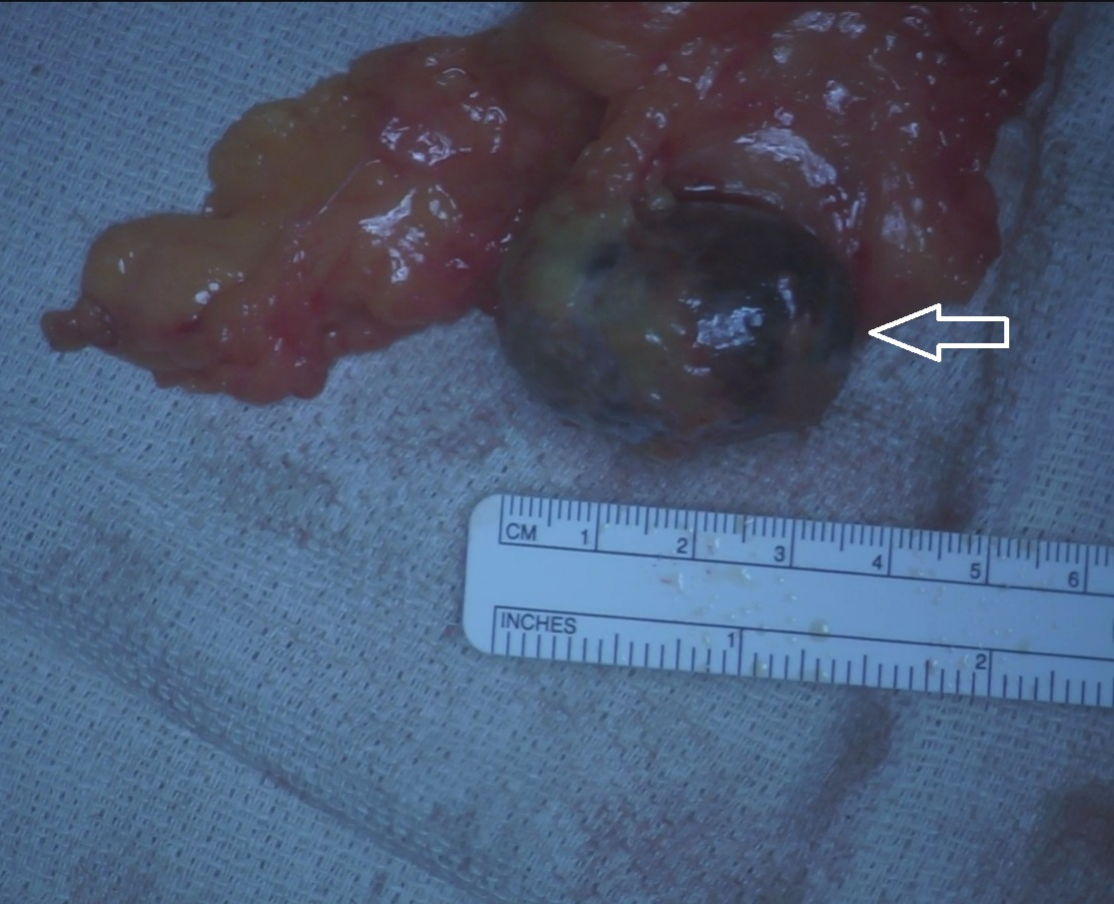
Intraoperative photo showing intra-abdominal mass (arrow).

Pathology reported an 8.7 x 7.5 x 2.8 cm irregular segment of serosal covered, lobulated, yellow-pink adipose tissue consistent with lipoma and a 3.9 x 2.7 x 2.3 cm dark brown, ovoid apparent calculus embedded within membranous tissue associated with foreign body reaction and chronic inflammation (Figure 2).

Postoperatively the patient had an uneventful recovery and was discharged on postoperative day zero. On her two weeks postoperative visit, she was doing well and had no acute concerns.

## Discussion

The complications of retained peritoneal gallstones are usually associated with delayed presentation of unexpected clinical problem. There is often a mean time of 4.8 months between onset of symptoms and the diagnosis of retained gallstone being made [1]. CT scan and ultrasound scan are the most useful diagnostic tests [1]. Having a high index of suspicion is the key to early diagnosis. Patients are mostly asymptomatic, however, when symptomatic they may present with fever, cellulitis, intra-abdominal abscess, abdominal pain, jaundice, intra-abdominal mass, ileus, and intestinal obstruction [1].

The natural history of peritoneal gallstone is for local inflammatory response to cause it to be walled off by omentum and local fibrosis with partial reabsorption sometimes occurring [1]. Pigmented stones are more likely to be infected than cholesterol stones, with up to 80-90% of pigment stone containing bacteria such as *Escherichia coli, Klebsiella pneumoniae, *and* Enterococcus* [1].

Predisposing risk factors for complications after gallstone spillage include acute cholecystitis with infected bile, pigment gallstones, multiple stones (more than 15), gallstone size greater than 1.5 cm, elderly patients, male sex, and subphrenic/subhepatic location of retained gallstone [1-3].

Retained peritoneal gallstones are not without complications. The effort to minimize iatrogenic gallbladder perforation is important including using the surgical technique that minimizes the risk of gallbladder perforation. This includes establishing the correct plane in dissecting the gallbladder from the liver, minimizing diathermy injury, and having a low threshold for using hydrodissection in Calot’s triangle [1]. If a perforation occurs, attempt should be made to close the hole using the grasp forceps or by endoclip or endoloop; the use of suction devices to minimize the spilled bile and gallstones as well as the use of an endo-bag is mandatory [3]. However routine conversion to laparotomy for stone retrieval is not recommended [3-5].

Intraoperative management of spillage gallstones in the abdominal cavity involves immediate judicious removal of as many stones as possible. Thorough irrigation and suction should be performed after collecting the visible stones in order to minimize the number of lost stone. Clear documentation of the intraoperative gallstone spillage in the operative report is recommended for alerting clinician in the future to the possibility of retained stone causing any subsequent problems that might lead to a prompt diagnosis [2,3].

The complications of retained peritoneal gallstones are usually associated with delayed presentation of unexpected clinical problem. There is often a mean time of 4.8 months between onset of symptoms and the diagnosis of retained gallstone being made [1]. CT scan and ultrasound scan are the most useful diagnostic tests [1]. Having a high index of suspicion is the key to early diagnosis.

In our case, pathology was consistent with a large 4 cm gallstone encased in the omentum. The patient had a gallstone dropped into the abdomen at the time of her cholecystectomy years ago, and this became encased in omentum and developed a calcified rim around it. No malignant pathology was noted.

## Conclusions

The complications of retained peritoneal gallstones are infrequent. Having a high index of suspicion is the key to early diagnosis. Every effort should be made to remove spilled gallstones at the index operation to prevent future complications, however, conversion from laparoscopy to laparotomy for retrieval of spilt gallstone is not recommended.

## References

[1] Woodfield JC, Rodgers M, Windsor JA (2004). Peritoneal gallstones following laparoscopic cholecystectomy: incidence, complications, and management. Surg Endosc.

[2] Carmichael SP 2nd, Zwischenberger BA, Bernard AC (2014). Late reoperation for retained gallstone after laparoscopic cholecystectomy. Surg Laparosc Endosc Percutan Tech.

[3] Zehetner J, Shamiyeh A, Wayand W (2007). Lost gallstones in laparoscopic cholecystectomy: all possible complications. Am J Surg.

[4] Stock CT, O'Donnell T, Barie PS (2012). Retained gallstone after laparoscopic cholecystectomy. Surg Infect (Larchmt).

[5] Demirbas BT, Gulluoglu BM, Aktan AO (2015). Retained abdominal gallstones after laparoscopic cholecystectomy: a systematic review. Surg Laparosc Endosc Percutan Tech.

